# The role of socio-economic and lifestyle factors in hearing function in middle-aged adults

**DOI:** 10.1371/journal.pone.0344788

**Published:** 2026-04-03

**Authors:** Inez Sharp, Dialechti Tsimpida, Meher Lad, Helen E. Nuttall, Kate Slade

**Affiliations:** 1 Department of Psychology, Faculty of Science and Technology, Lancaster University, Bailrigg, United Kingdom; 2 Division of Public Health and Epidemiology, College of Life Sciences, University of Leicester, Leicester, United Kingdom; 3 Department of Gerontology, Faculty of Social Sciences, University of Southampton, Southampton, United Kingdom; 4 Faculty of Medical Sciences, Newcastle University, Newcastle upon Tyne, United Kingdom; 5 Lancaster Medical School, Faculty of Health and Medicine, Lancaster University, Bailrigg, United Kingdom; Universiti Malaya Fakulti Perubatan: University of Malaya Faculty of Medicine, MALAYSIA

## Abstract

**Objectives:**

This study aimed to investigate the associations between socioeconomic and lifestyle factors and measures of hearing ability to better understand potential risk factors for hearing loss. Insights from this research could help audiologists identify high-risk demographics and ultimately contribute to addressing inequalities in hearing health.

**Design:**

An online study design was used, where data were collected on participant demographics, lifestyle and socioeconomic status, including age, gender, ethnicity, region of residence, income, education, occupation, exercise frequency, height and weight, smoking status, and weekly alcohol consumption, which were used as model predictors. Participants also self-reported their hearing function using the Speech, Spatial and Qualities of Hearing Scale 12 (SSQ-12) and completed an online digits-in-noise (DiN) task to assess speech perception ability, which were used as outcome variables. A sample of 227 adults aged 45–65 (mean age = 53.77, SD = 5.87) were recruited through Prolific (www.prolific.com) based on Office for National Statistics (ONS) income groups.

**Results:**

Two multiple regression models were conducted with the outcome variables of speech perception ability (Digits-in-Noise: DiN) and self-reported hearing function (Speech and Spatial Qualities of Hearing Scale: SSQ-12). The analysis revealed that having a routine and manual occupation predicted worse self-reported hearing function, as did being a regular tobacco consumer. However, no predictors were significantly associated with speech perception ability.

**Conclusions:**

The findings suggest that socioeconomic and lifestyle factors, as measured in this study, are significantly associated with self-reported hearing function in mid-life, but not with speech in noise perception ability. These results partially align with previous research showing that socioeconomic and lifestyle factors, including smoking and occupation, are associated with hearing sensitivity, when measured using pure tone audiometry. These data highlight the need for greater understanding as to how lifestyle and socioeconomic factors relate to different dimensions of hearing health.

## Introduction

Hearing loss is frequently accepted as an unavoidable consequence of ageing. However, not everyone experiences age-related hearing loss (ARHL); in fact, the validity of the concept of age-related hearing loss has been questioned [1]. Lifestyle-related hearing loss may be a more accurate conceptualisation; the accumulation of lifestyle factors over time may be the mechanism behind age-related declines. It may be the case that modifiable lifestyle factors and health inequalities lead to an increased likelihood of hearing loss in older age. Lifestyle factors may include modifiable factors such as diet, physical activity, noise exposure, or body mass index; however, lifestyle may be strongly impacted by non-modifiable factors such as geographical location [2], cultural norms and socioeconomic position [1]. For example, living in an area where there is limited access to affordable healthy foods and recreational spaces will reduce the modifiability of lifestyle choices.

Hearing loss is not only a challenging sensory deficit; it impacts quality of life, and is related to reduced wellbeing [3], increased social isolation [4] and depression [5]. Previous data have indicated that the socioeconomic position of participants influenced the strength of the relationship between hearing loss and depression, and people with lower income had up to double the relative risk for depression compared to their counterparts with higher income [5]. Understanding the socioeconomic and lifestyle factors which contribute to hearing difficulty in later life, will highlight avenues for intervention and reduce the burden of preventable hearing loss on both the individual and the healthcare system.

Previous research highlights that lower socioeconomic position (SEP; an umbrella term which often incorporates education, occupation, income and wealth factors), and lifestyle variables (including body mass index, physical inactivity, smoking and alcohol consumption) are associated with poorer hearing, as strongly as age and gender [1]. The link between SEP, lifestyle factors, and hearing loss is multifaceted [6]. SEP factors contribute to social and health inequality [6]. Such factors increase the likelihood of high-noise exposure occupations [7]; financial barriers to audiological treatments [1]; financial stresses which may be associated with unhealthy eating, e.g., eating lower cost food that is less nutritionally rich, increased smoking, and alcohol consumption [6,8]. Unhealthy eating could be predictive of hearing loss due to dietary behaviours that reduce vitamin B12 levels [9]. B12 plays an important role in cell metabolism, vascular function, and myelin synthesis; the impairment of these functions may increase the risk of hearing loss [10]. Similarly, the association between alcohol consumption and hearing loss may be due to alcohol induced B12 depletion [11]. The association between smoking and hearing loss may be due to oxidative stress, an imbalance of free radicals and antioxidants in the body [12]. Due to the high metabolic demands of the cochlea while responding to stimuli, the mitochondria of hair cells produce high levels of reactive oxygen species. This can result in cochlear degeneration when the antioxidants of hair cells are compromised by external factors, such as smoking, which may result in hearing loss [13–16]. All these factors increase the likelihood of poorer hearing. Critically, SEP disparities cultivate inequitable situations in which prioritising behaviours for hearing health is not an option for a proportion of the population, such as health-seeking behaviours like taking precautions to reduce noise exposure and accessing audiological services.

The present study investigates how SEP and lifestyle factors impact hearing ability and speech perception in middle-aged adults aged 45–65 years old. Previous research has focused on hearing sensitivity measured using pure tone audiometry [1], but communication in naturalistic settings requires understanding suprathreshold stimuli, such as speech in noise. Difficulties with speech perception in noisy environments are a common complaint, and such problems may be a precursor to clinical hearing loss [17]. This research aims to determine which, if any, SEP and lifestyle factors affect speech in noise perception ability and subjective hearing ability in middle age. Building on previous research that measured hearing function using one dimension, hearing sensitivity [1], this research measures two not yet explored dimensions of hearing function, speech perception ability and subjective hearing ability. Furthermore, this research targets a socioeconomically representative sample, recruited via income groups; previous research has utilised large-scale volunteer datasets such as The English Longitudinal Study of Ageing (ELSA) that have disproportionately high dropout rates for lower socioeconomic groups [18]. Identifying the factors which may predict reduced hearing function in mid-life will allow for early implementation of lifestyle interventions to address risk factors, encourage uptake of hearing aids in at-risk populations, and reduce hearing health inequalities, as audiologists can target groups in most need.

Specifically, this study aimed to explore the role of socioeconomic position (incorporating level of education, occupation, and income), demographic factors that relate to socioeconomic position (including region of residence, ethnicity, age, and gender), and health-related lifestyle factors (including body mass index, physical activity, smoking status, and alcohol consumption), in speech perception ability and self-reported hearing ability in middle-aged adults (45–65 years).

## Methods

Ethical approval was obtained from Lancaster University’s Faculty of Science and Technology Ethics Committee (FST-2022–0790-MA-1), and the research study was preregistered on the Open Science Framework (https://osf.io/xju76).

### Participants

The participants included 227 adults (110 males and 117 females) aged between 45–65 (*M* = 53.78, *SD* = 5.87). The number of participants recruited was based on an a-priori power analyses using G*Power [19], which suggested a minimum sample size of 124 participants. The minimum requirement was calculated by inputting 11 predictors, setting alpha at .05, power at 80%, and Cohen’s *f*^2^ to .15, representing a medium effect size. Further participants were recruited to ensure as much equal distribution across income bands as possible, this was facilitated by Prolific’s [20] pre-screening function, allowing balanced recruitment from different income bands. All participants resided in the UK, did not have an ear infection at the time of the study, did not have a cochlear implant and self-reported having used headphones for the speech perception task. The participants were recruited based on Office for National Statistics (ONS) income distribution groups: £0-£12,999 (N = 31), £13,000-£20,499 (N = 37), £20,500-£26,799 (N = 29), £26,800-£35,699 (N = 37), £35,700-£53,999 (N = 41), > £54,000 (N = 52), to facilitate a wide distribution of SEP. Participants were recruited using the online portal Prolific [20] and were paid £5 on completion of the study.

### Materials

#### Predictor variables.

##### Gender:

Gender was measured as a self-reported categorical variable (female, male, non-binary).

##### Age:

Age was measured on a continuous scale; participants were given a drop-down box with ages 45–65 to select from.

##### Region of residence:

Region of residence in the UK was measured as a self-reported categorical variable. Participants could select from the options: Scotland, Northern Ireland, North-East, North-West, Yorkshire and the Humber, East Midlands, West Midlands, Wales, East of England, London, South-East, and South-West. The region categories were based on the international territorial levels [21] and reflect the categories used by Tsimpida et al. [22].

##### Ethnicity:

Ethnicity was recorded from participants’ self-reported ethnic group; the categories were selected from the 13 categories provided in the UK Governmental Statistical Service (GSS) ethnicity harmonised standard [23].

##### Body Mass Index (BMI):

BMI was calculated from participants’ self-reported height and weight according to the equation: BMI = kg/m2, where kg is the person’s weight in kilograms and m2 is their height in meters squared. Being underweight was categorised as a BMI < 18.5, healthy weight as 18.5–24.99, overweight as 25–29.99, and obese as >30.

##### Level of physical activity:

Level of physical activity was measured by asking participants to rate on a four-point scale how often they complete moderate-to-high-intensity physical activity (e.g., activities like brisk walking, riding a bike, mowing the grass). Response options included (1) more than once a week, (2) once a week, (3) one to three times a month and (4) hardly ever or never. The categories reflect those used by Tsimpida et al. [1].

##### Tobacco consumption:

Tobacco consumption was measured by asking participants to disclose their consumption of Tobacco products. Response options included (1) regular, current smoker/consumer (2) occasional current smoker/consumer (3) former smoker/consumer (4) never smoked/consumed.

##### Levels of alcohol consumption:

Levels of alcohol consumption were self-reported by participants. Participants were asked to disclose their alcohol intake in units over the last 7 days. To help participants respond accurately, they were provided with guidance on the number of alcohol units in a range of common alcoholic beverages.

##### Occupation:

Occupation was measured as a self-reported categorical variable. Participants were asked to select the occupation they have spent the most years on. The 2020 UK Standard Occupational Classification major group structure [24] was used to create the ten response categories. Examples of jobs that fit in each major category were provided to support participants’ accuracy.

##### The highest level of educational attainment:

The highest level of educational attainment was self-reported by participants. Participants selected from the options: no qualifications, foreign or other, O level Certificate of Secondary Education or GCSE, A level (or level 3 equivalent), degree or higher education. The categories reflect those used by Tsimpida et al. [1,22].

##### Net household income:

Net household income was measured by asking participants to select their net household income from 6 categories. The categories were provided according to ONS income distribution, containing the average (median) annual household income by quintile from the year ending March 2019 [25]. The options were: above £54,000, between £35,700 and £54,000, between £26,800 and £35,699, between £20,500 and £26,799, between £13,000 and £20,499, or below £13,000.

#### Outcome variables.

##### Speech, Spatial and Qualities of Hearing Scale-12 (SSQ-12):

The SSQ-12 [26] is a questionnaire that enquires about an individual’s perceptions of their hearing ability and hearing quality in different lifestyle situations. The questionnaire is used to measure subjective hearing function. Participants responded on a 10-point Likert scale where 0 indicated that they would be unable to hear or listen in the situation described, and 10 indicated that they would be perfectly able to hear or listen in the described situation. Therefore, scores ranged from 12 (perfect hearing) to 0 (poorest hearing). An example item is, ‘You are talking with one other person, and there is a TV on in the same room. Without turning the TV down, can you follow what the person you’re talking to says?’. The scores are averaged over all items to create a mean SSQ-12 score; lower mean scores indicate poorer self-reported hearing ability.

##### Remote Digits-in-Noise Task:

The remote Digits-in-Noise (DiN) task measures speech perception accuracy [27]. It presents three numbers via an audio recording of a female voice with varying signal-to-noise ratios. During the DiN task, a participant hears three numbers and reports the numbers they heard using an onscreen keypad. An adaptive 1-up, 1-down psychophysical paradigm was implemented whereby a correct response resulted in the speech-to-noise ratio (SNR) being reduced and an incorrect one caused the SNR to increase, both the speech and noise were adapted. The starting SNR was 0 dB and the step sizes decreased from 5 to 2 dB after 3 reversals, which then reduced to 0.5 dB after 3 more reversals. There were no familiarisation trials as to avoid participant fatigue, however there was a presentation of digits on a background of noise at the starting SNR that could be played as many times as the user needed to feel comfortable with hearing the sound before starting the task. The run terminated after 10 reversals and the SNR at the last 5 reversals was averaged to calculate the DIN threshold (SNR-50, the signal to noise ratio at which the participant achieved 50% accuracy) for each participant. A lower SNR-50 is related to better speech perception ability, whereas a higher SNR-50 indicates poor speech perception ability.

### Procedure

Participants accessed the study through Prolific [20] using pre-screening filters for age, income, country of residence and cochlear implant use. They were redirected to an online Qualtrics questionnaire; they used their phones or computers and could complete the survey in their chosen location. All participants were provided with identical questionnaires. Before access was given to the questionnaire, participants provided their informed consent. They then had to enter their unique prolific ID for subsequent renumeration. After this, they were directed to a set of screening questions; they were screened for age, hearing disorders and UK residency. If they did not meet the inclusion criteria, participants were redirected to the end of the survey and the debrief sheet. The participants who did meet the inclusion criteria were directed to the self-reported lifestyle factors survey, the SSQ-12 [26] and the DiN task. Prolific encourages the use of attention checks and allows for participant data to be rejected if they have failed two or more attention checks [28]. Within the survey there were three attention checks, two of which were instructional manipulation tasks. Participants were asked, “The test you are about to take part in is very simple; when asked for your favourite country, you must select ‘Thailand’. This is an attention check. Based on the text you read above, what country have you been asked to select?”. Participants could select from ‘India’, ‘Cambodia’, ‘Bangladesh’, ‘Thailand’ and ‘Vietnam’. The second attention check asked: “The Maths test you are about to take part in is very simple; when asked what is ‘2+2’, you must answer ‘5’. This is an attention check. Based on the text you read above, which answer have you been asked to select?”. The third attention check was a nonsensical item, the statement “The moon is made out of cheese” was paired with a Likert scale, to pass the check participants had to select “strongly disagree” or “disagree”. No participants failed the attention checks. Participants were required to wear headphones for the DiN task. To check this, participants were asked to self-report if they were wearing headphones and to indicate the type of headphones they were wearing, as well as the type of device they were using, i.e., mobile or laptop. No participants reported being hearing aid users. After completing the DiN task, participants were debriefed and redirected to Prolific for renumeration.

### Statistical analysis

Descriptive statistics for all planned analyses were reported. Data were analysed using multiple linear regression models. Two separate regression models were run to determine which variables best predict subjective hearing ability and speech perception performance. The outcome variables in the two multiple regression models were: 1) subjective hearing ability, as quantified by the mean score on the Speech and Spatial Qualities of Hearing Scale (SSQ-12 [26]); 2) speech perception performance, as quantified by the SNR-50 (the signal-to-noise ratio at which a participant achieves a 50% performance standard) obtained from an online Digits in Noise (DiN) task. The predictor variable, region, was grouped into three categories: north, midlands and south. Occupation was categorised into four categories: unemployed, routine and manual, intermediate, and professional and managerial occupations. The predictor, educational attainment, was dichotomised into those with a degree or higher education and those without a degree or higher education. The alcohol consumption predictor was dichotomised into those who consumed more than 14 units of alcohol and those who consumed less than 14 units of alcohol in the last 7 days. Table 1 displays the transformations of the collapsed variables. The transformations were made to simplify the models and increase their interpretability. Gender was dichotomised into male and female, as there were no non-binary participants. Age was maintained as a continuous variable.

**Table 1:** Table displaying the raw counts of and recoding of collapsed variables, Region, Occupation and Education level.

Collapsed variables	Original categorisations
**Region**	
South (N = 79)	South East (N = 36), South West (N = 19), London (N = 24)
Midlands (N = 77)	East Midlands (N = 19), East of England (N = 27), West Midlands (N = 22), Wales (N = 9)
North (N = 71)	North West (N = 23), North East (N = 7), Yorkshire and the Humber (N = 20), Scotland (N = 17), Northern Ireland (N = 4)
**Occupation**	
Managerial and professional (N = 69)	Managers, directors and senior officials (N = 24), Professional occupations (N = 45)
Intermediate (N = 105)	Associate professional and technical occupations (N = 32), Administrative occupations (N = 59), Skilled trades occupations (N = 14)
Routine and manual (N = 46)	Caring, leisure and other service occupations (N = 13), Elementary occupations (N = 6), Process, plant and machine operatives (N = 5), Sales and customer service occupations (N = 22)
Unemployed (N = 7)	Unemployed (N = 7)
**Education level**	
Degree or higher education (N = 128)	Degree or higher education (N = 128)
No degree (N = 99)	A level (or level 3 equivalent) (N = 44), O level Certificate of Secondary Education or GCSE (N = 50), Foreign or other (N = 3), No qualifications (N = 2)

For both the models predicting self-reported hearing ability (SSQ-12) [26] and speech perception ability (SNR-50) the included predictors were: Alcohol consumption (Less than vs More than 14 units per week), Age (Years), BMI group (Healthy vs Overweight vs Obese vs Underweight), Gender (Male vs Female), Education (Degree or higher vs No degree) Income (£0-£12,999 vs £13,000-£20499 vs £20,500-£26,799 vs £26,800-£35,699 vs £35,700-£53,999 vs >£54,000), Occupation (Routine and Manual vs Intermediate vs Managerial and Professional vs Unemployed), Exercise (Less than once a week vs Once a week vs Less than once a week), Region (North vs Midlands vs South), Tobacco consumption (Current smoker vs Former smoker vs Never smoked).

### Deviations from pre-registration

The ethnicity predictor was removed from the analyses due to a lack of variance in the sample (White: N = 216, All other ethnic groups combined: N = 11). The pre-registered analysis plan was changed to omit the use of stpwise model selection with the StepAIC() function from the R MASS package [29] due to concerns about the reliability of stepwise logistic regressions when attempting to determine risk factors for medical illnesses [30]. Austin and Tu conducted 1,000 runs of backward elimination on one dataset and produced 940 different “optimal” models, suggesting the instability of stepwise model selection [31]. Therefore, only the full models were reported. To address possible type I error inflation, the Family-Wise Error Rate was controlled through Bonferroni correction. As such, the criterion for statistical inference was modified by dividing the alpha level of .05 by the number of tested hypotheses, (.05/9), which provided a new inference criteria of *p* = .005.

## Results

### Data analysis

**Fig 1:**
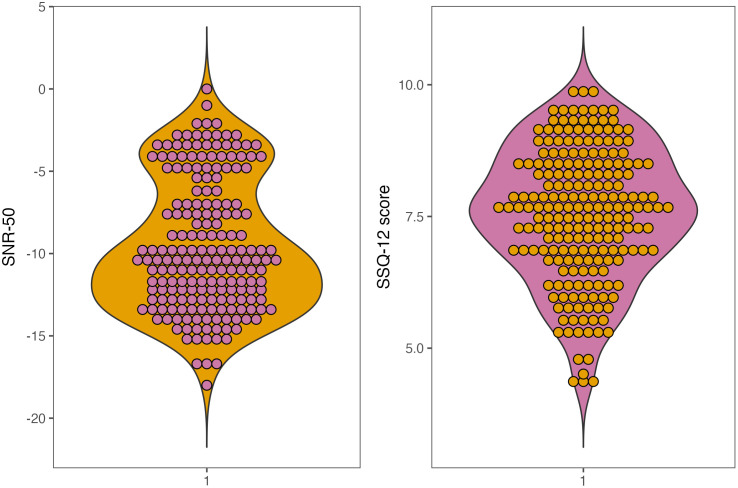
Violin plots displaying the data distributions for SNR-50 and SSQ-12 scores when influential data points are removed.

Data points were removed if they were more than three standard deviations away from the mean SSQ-12 or SNR-50 [32]. Two participants were excluded based on having an outlying SNR-50. Influential outliers were investigated using Cook’s distance; data points > 4/n were classified as outliers and were removed from the models (n is the number of data points, i.e., the sample size). For the speech perception accuracy model, 13 influential data points were removed; for the subjective auditory function model, 18 influential data points were removed. The data distributions without influential data points for speech perception accuracy and subjective auditory function are displayed in Fig 1.

Data pre-processing and analyses were conducted in R Studio [33]. We utilised the packages tidyverse and dpylr [34,35], the car package [36], ggthemes and ggpubr [37,38] for data management and visualisation; effect sizes were computed using the rsq package [39].

#### Speech perception accuracy.

**Fig 2:**
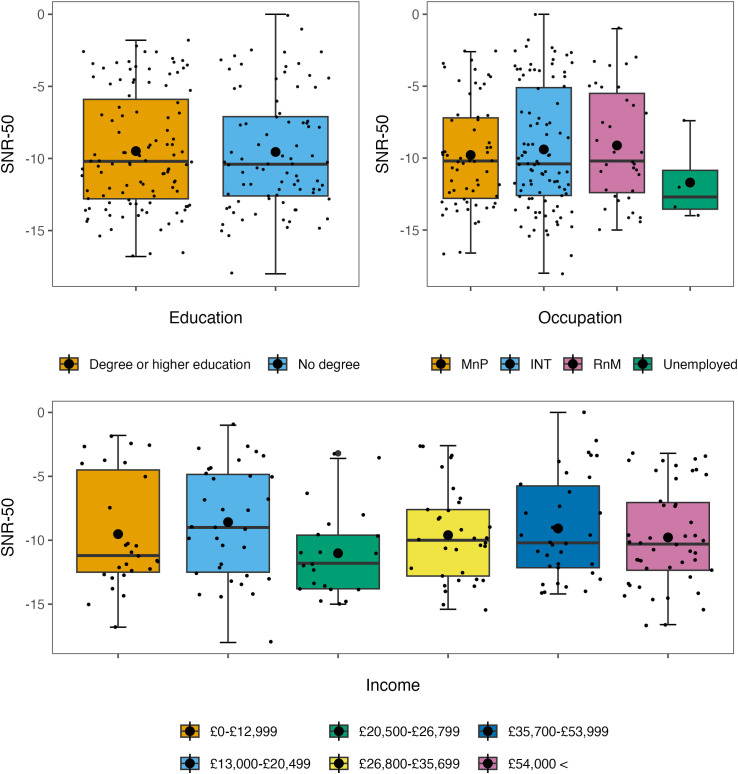
Box plots showing the distributions of SNR-50 when stratified by socioeconomic factors. Note. The larger black dots represent the mean SNR-50 for each group; the smaller black dots represent individual data points. MnP = managerial and professional occupation, INT = intermediate occupation, RnM = routine and manual occupation.

The mean SNR-50 was −9.3 (*SD* = 4.37); a lower SNR-50 reflects better hearing. The model without influential data points removed is reported (Table 2). The model was non-significant overall, *R*^2^_*adjusted*_ = −.05, *F*(23, 184) = 0.61, *p* = .92. The model with influential data points removed is reported (Table 3). The model was non-significant overall, *R*^2^_*adjusted*_ = 0.02, *F*(23, 171) = 1.14, *p* = .31. The distributions of SNR-50 when stratified by socioeconomic status are reported in Fig 2.

**Table 2:** Linear multiple regression model predicting speech perception accuracy (SNR-50) before influential outlier removal.

SNR-50 ~ Age + Alcohol consumption + BMI group + Gender + Education level + Income + Occupation + Exercise frequency + Region of residence + Tobacco consumption					
	β	SE	*t*	*p*	partial R²
**Constant**	−10.73	3.24	−3.24	.001	
**Age**	0.01	0.06	0.19	.848	.0002
**Alcohol consumption**					.003
Over 14 units (N = 34)	0.69	0.91	0.75	.454	
*Under 14 units (reference) (N = 174)*					
**BMI group**					.03
Overweight (N = 65)	−1.02	0.77	−1.33	.184	
Obese (N = 52)	0.71	0.84	0.81	.396	
Underweight (N = 4)	2.64	2.33	1.13	.260	
*Healthy weight (reference) (N = 87)*					
**Gender**					.001
Male (N = 106)	0.03	0.67	0.45	.652	
*Female (reference) (N = 102)*					
**Education level**					.001
No degree (N = 89)	−0.27	0.74	−0.37	.714	
*Degree or higher education (reference) (N = 119)*					
**Income**					.03
<£13,000 (N = 29)	1.30	1.14	1.14	.255	
£13,000-£20,499 (N = 35)	1.52	1.09	1.40	.162	
£20,500-£26,799 (N = 25)	0.01	1.21	0.01	.993	
£26,800-£53,699 (N = 33)	0.56	1.07	0.52	.602	
£35,700-£53,999 (N = 37)	1.87	1.02	1.83	.070	
*>£54,000 (reference) (N = 49)*					
**Occupation**					.01
Intermediate (N = 98)	0.45	0.80	0.57	.571	
Routine and Manual (N = 38)	1.07	1.11	0.97	.333	
Unemployed (N = 6)	−0.53	2.02	−0.26	.794	
*Managerial and Professional (reference) (N = 66)*					
**Exercise frequency**					.02
Hardly ever or never (N = 23)	−1.28	1.12	−1.14	.254	
One to three times a month (N = 9)	0.71	1.63	0.43	.665	
Once a week (N = 44)	−1.23	0.84	−1.46	.146	
*More than once a week (reference) (N = 132)*					
**Region of residence**					.0004
Midlands (N = 71)	0.21	0.80	0.26	.792	
North (N = 67)	0.18	0.82	0.23	.822	
*South (reference) (N = 70)*					
**Tobacco consumption**					.01
Former smoker/consumer (N = 54)	−0.73	0.81	−0.91	.366	
Occasional smoker/consumer (N = 8)	0.48	1.74	0.28	.783	
Regular smoker/consumer (N = 51)	−0.28	0.82	−0.34	.732	
*Never smoked/consumed (reference) (N = 95)*					

*Note.* β = Regression coefficient, SE = Standard error.

**Table 3:** Linear multiple regression model predicting speech perception accuracy (SNR-50) after influential outlier removal.

SNR-50 ~ Age + Alcohol consumption + BMI group + Gender + Education level + Income + Occupation + Exercise frequency + Region of residence + Tobacco consumption					
	β	SE	*t*	*p*	partial R^2^
**Constant**	−14.21	3.00	−4.73	**<.001**	
**Age**	0.09	0.05	1.60	.111	.02
**Alcohol consumption**					.01
Over 14 units (N = 33)	0.91	0.85	1.07	.287	
*Under 14 units (reference) (N = 162)*					
**BMI group**					.05
Overweight (N = 59)	−1.34	0.71	−1.89	.061	
Obese (N = 49)	0.78	0.76	1.03	.303	
Underweight (N = 1)	5.36	4.13	1.30	.196	
*Healthy weight (reference) (N = 86)*					
**Gender**					.0004
Male (N = 99)	−0.16	0.63	−0.25	.802	
*Female (reference) (N = 96)*					
**Education level**					.004
No degree (N = 84)	−0.57	0.71	−0.80	.426	
*Degree or higher education (reference) (N = 111)*					
**Income**					.04
<£13,000 (N = 27)	0.57	1.07	0.54	.591	
£13,000-£20,499 (N = 34)	1.67	1.00	1.67	.097	
£20,500-£26,799 (N = 21)	−0.94	1.17	−0.80	.424	
£26,800-£53,699 (N = 33)	0.58	0.97	0.60	.552	
£35,700-£53,999 (N = 34)	1.27	0.96	1.32	.190	
*>£54,000 (reference) (N = 46)*					
**Occupation**					.02
Intermediate (N = 95)	0.87	0.75	1.16	.249	
Routine and Manual (N = 35)	1.58	1.04	1.52	.129	
Unemployed (N = 4)	−0.40	2.18	−0.18	.857	
*Managerial and Professional (reference) (N = 61)*					
**Exercise frequency**					.03
Hardly ever or never (N = 22)	−1.84	1.03	−1.80	.074	
One to three times a month (N = 8)	−0.82	1.55	−0.53	.597	
Once a week (N = 42)	−1.11	0.76	−1.47	.014	
*More than once a week (reference) (N = 123)*					
**Region of residence**					.001
Midlands (N = 68)	0.27	0.73	0.37	.715	
North (N = 62)	0.12	0.76	0.16	.872	
*South (reference) (N = 65)*					
**Tobacco consumption**					.04
Former smoker/consumer (N = 47)	−1.88	0.77	−2.46	.**015**	
Occasional smoker/consumer (N = 6)	0.37	1.78	0.21	.837	
Regular smoker/consumer (N = 51)	−0.39	0.75	−0.53	.598	
*Never smoked/consumed (reference) (N = 91)*					

*Note.* β = Regression coefficient, SE = Standard error

#### Subjective auditory function.

**Fig 3:**
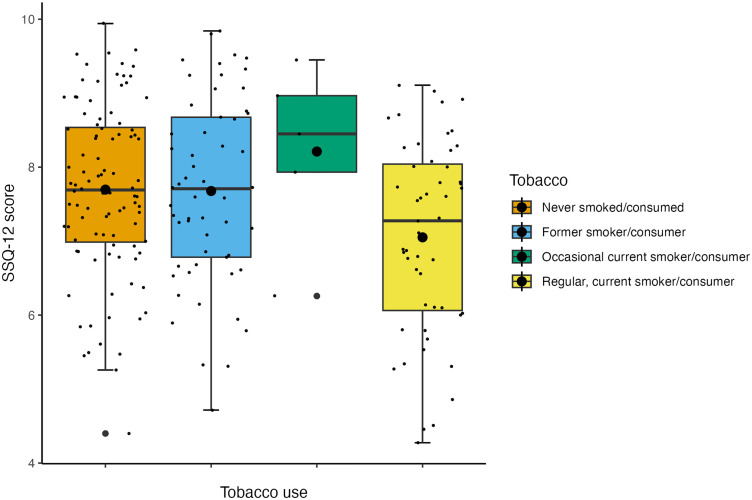
Box plot displaying the effect of tobacco consumption on subjective auditory function after influential outlier removal. *Note.* The larger black dots represent the mean SSQ-12 score for each group; the smaller black dots represent individual data points.

**Fig 4:**
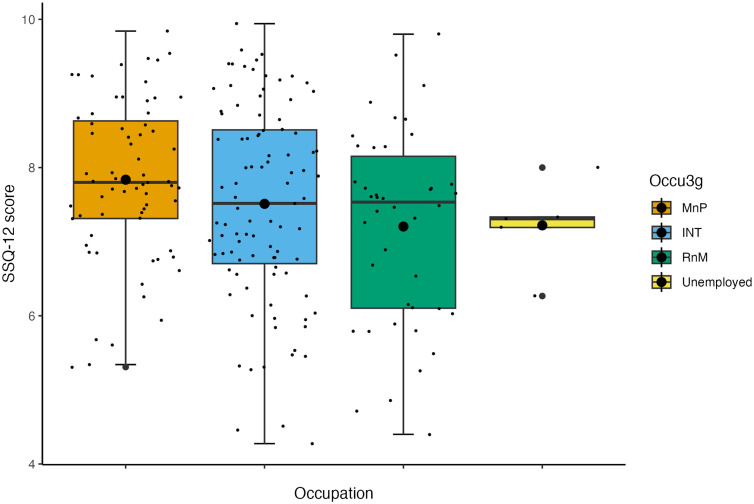
Box plot displaying the effect of occupation on subjective auditory function after influential outlier removal. Note. The larger black dots represent the mean SSQ-12 score for each group; the smaller black dots represent individual data points. MnP = managerial and professional occupation, INT = intermediate occupation, RnM = routine and manual occupation.

**Fig 5:**
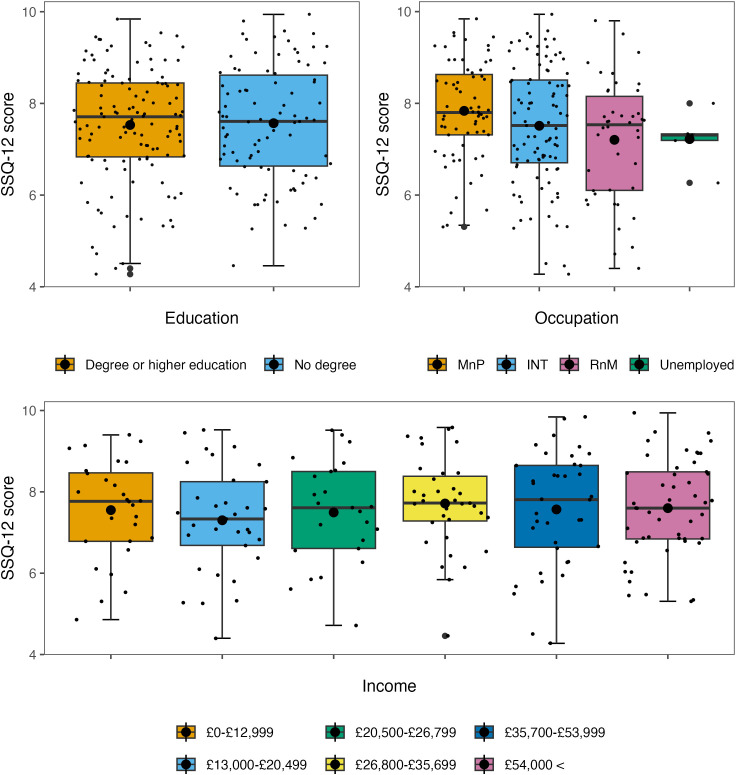
Box plots showing the distribution of SSQ-12 scores when stratified by socioeconomic factors. Note. The larger black dots represent the mean SSQ-score for each group; the smaller black dots represent individual data points. MnP = managerial and professional occupation, INT = intermediate occupation, RnM = routine and manual occupation.

The internal consistency of the SSQ-12 [26] was measured by calculating Cronbach’s alpha, *α* = .89. And the Item-Rest Correlations for the 12 items ranged from *r* = .40 to .78. The mean SSQ-12 score was 7.47 (*SD* = 1.45), a higher SSQ-12 score reflects better-self-reported hearing: Scores can range from 0–10. The model without influential data points removed is reported (Table 4). The model was non-significant overall *R*^2^_*adjusted*_ = 0.01, *F*(23, 203) = 1.07, *p* = .379. The model with influential data points removed is reported (Table 5). The model was non-significant overall, *R*^2^_*adjusted*_ = 0.10, *F*(23, 185) = 1.97, *p* = .007. The model with influential data points removed found that being a regular tobacco consumer significantly predicted worse self-reported auditory function compared to those who had never smoked/consumed (β = −0.74, *p* <  .001) (Fig 3) and that having a routine and manual occupation compared to having a managerial and professional occupation significantly predicted worse self-reported auditory function (β = −1.01, *p* <  .001) (Fig 4). The distributions of SNR-50 when stratified by socioeconomic status are reported in Fig 5.

**Table 4:** Linear multiple regression model predicting subjective auditory function (SSQ-12 score) before influential outlier removal.

SSQ-12 score ~ Age + Alcohol consumption + BMI group + Gender + Education level + Income + Occupation + Exercise frequency + Region of residence + Tobacco consumption					
	β	SE	*t*	*p*	partial R^2^
**Constant**	8.92	0.98	9.07	**<.001**	
**Age**	−0.02	0.02	−1.01	.315	.01
**Alcohol consumption**					.0002
Over 14 units (N = 38)	0.05	0.28	0.19	.853	
*Under 14 units (reference) (N = 189)*					
**BMI group**					.01
Overweight (N = 70)	0.08	0.24	0.33	.741	
Obese (N = 59)	−0.32	0.25	−1.27	.206	
Underweight (N = 4)	0.07	0.75	0.09	.931	
*Healthy weight (reference) (N = 94)*					
**Gender**					.01
Male (N = 110)	−0.32	0.21	−1.54	.124	
*Female (reference) (N = 117)*					
**Education level**					.004
No degree (N = 99)	0.20	0.23	0.85	.397	
*Degree or higher education (reference) (N = 128)*					
**Income**					.01
<£13,000 (N = 31)	0.15	0.35	0.43	.671	
£13,000-£20,499 (N = 37)	−0.09	0.34	−0.25	.800	
£20,500-£26,799 (N = 29)	−0.06	0.37	−0.17	.864	
£26,800-£53,699 (N = 37)	0.18	0.33	0.55	.584	
£35,700-£53,999 (N = 41)	−0.04	0.32	−0.13	.897	
*>£54,000 (reference) (N = 52)*					
**Occupation**					.03
Intermediate (N = 105)	−0.38	0.25	−1.55	.122	
Routine and Manual (N = 46)	−0.89	0.34	−2.63	**.009**	
Unemployed (N = 7)	−0.53	0.62	−0.87	.388	
*Managerial and Professional (reference) (N = 69)*					
**Exercise frequency**					.01
Hardly ever or never (N = 24)	−0.02	0.35	−0.07	.946	
One to three times a month (N = 10)	−0.26	0.50	−0.52	.606	
Once a week (N = 48)	−0.24	0.26	−0.92	.358	
*More than once a week (reference) (N = 145)*					
**Region of residence**					.01
Midlands (N = 77)	0.11	0.24	0.45	.651	
North (N = 71)	0.37	0.25	1.46	.146	
*South (reference) (N = 79)*					
**Tobacco consumption**					.03
Former smoker/consumer (N = 60)	0.08	0.25	0.31	.759	
Occasional smoker/consumer (N = 9)	−0.12	0.52	−0.23	.818	
Regular smoker/consumer (N = 56)	−0.55	0.25	−2.18	**.031**	
*Never smoked/consumed (reference) (N = 102)*					

*Note.* β = Regression coefficient, SE = Standard error

**Table 5:** Linear multiple regression model predicting subjective auditory function after influential outlier removal (SSQ-12 score).

SSQ-12 score ~ Age + Alcohol consumption + BMI group + Gender + Education level + Income + Occupation + Exercise frequency + Region of residence + Tobacco consumption					
	β	SE	*t*	*p*	partial R^2^
**Constant**	9.72	0.85	11.50	**<.001**	
**Age**	−0.03	0.02	−1.84	.068	.02
**Alcohol consumption**					.00003
Over 14 units (N = 35)	0.02	0.24	0.07	.946	
*Under 14 units (reference) (N = 174)*					
**BMI group**					.03
Overweight (N = 63)	0.01	0.21	0.04	.972	
Obese (N = 55)	−0.49	0.22	−2.23	**.027**	
Underweight (N = 3)	−0.55	0.71	−0.77	.455	
*Healthy weight (reference) (N = 88)*					
**Gender**					.02
Male (N = 103)	−0.34	0.18	−1.89	.061	
Female (reference) (N = 106)					
**Education level**					.02
No degree (N = 90)	0.38	0.21	1.81	.072	
*Degree or higher education (reference) (N = 119)*					
**Income**					.01
<£13,000 (N = 28)	0.12	0.31	0.39	.700	
£13,000-£20,499 (N = 33)	−0.10	0.30	−0.34	.734	
£20,500-£26,799 (N = 25)	−0.13	0.32	−0.40	.689	
£26,800-£53,699 (N = 35)	0.09	0.28	0.34	.733	
£35,700-£53,999 (N = 39)	0.03	0.27	0.10	.919	
*>£54,000 (reference) (N = 49)*					
**Occupation**					.06
Intermediate (N = 95)	−0.59	0.21	−2.74	**.007**	
Routine and Manual (N = 45)	−1.01	0.30	−3.39	**<.001**	
Unemployed (N = 5)	−0.63	0.60	−1.05	.297	
*Managerial and Professional (reference) (N = 67)*					
**Exercise frequency**					.01
Hardly ever or never (N = 21)	0.09	0.30	0.30	.764	
One to three times a month (N = 7)	−0.53	0.49	−1.08	.283	
Once a week (N = 45)	−0.21	0.22	−0.95	.343	
*More than once a week (reference) (N = 136)*					
**Region of residence**					.02
Midlands (N = 70)	0.12	0.21	0.64	.525	
North (N = 68)	0.37	0.21	1.76	.080	
*South (reference) (N = 71)*					
**Tobacco consumption**					.07
Former smoker/consumer (N = 57)	0.06	0.21	0.26	.792	
Occasional smoker/consumer (N = 5)	0.19	0.57	0.34	.736	
Regular smoker/consumer (N = 51)	−0.74	0.22	−3.37	**<.001**	
*Never smoked/consumed (reference) (N = 96)*					

*Note.* β = Regression coefficient, SE = Standard error

## Discussion

Potential socioeconomic and lifestyle predictors of midlife hearing function were investigated in this online study, with subjective hearing ability measured using the SSQ-12 [26] and speech perception measured with a remote DiN task (measuring SNR-50). Participants recruited through Prolific were recruited based on ONS income group, so there were equal participant numbers between socioeconomic bands.

When controlling for Family-Wise Error Rate through Bonferroni correction (dividing alpha by the number of tested hypotheses and using *p* < .005 as the statistical inference criteria) there were no significant relationships between lifestyle or socioeconomic factors and speech perception ability (regardless of whether influential data points were removed). In the model with influential data points removed, the predictors that had the largest effect sizes were income group (partial R² = .04) and tobacco consumption (partial R² = .04); these effect sizes are small-medium (.01 indicates a small effect size and .06 a medium effect size [40]) and represent a narrow picture of what predicts speech perception ability. There were, however, significant relationships between lifestyle and socioeconomic factors and self-reported hearing function. In the model with influential data points removed, having a routine and manual occupation as compared to having a managerial and professional occupation was significantly associated with worse subjective auditory function (*p* < .001). Regular tobacco consumption as compared to non-consumption was significantly associated with worse subjective auditory function (*p* < .001). The model predictors with the largest effect sizes were occupation (partial R² = .06) and tobacco consumption (partial R² = .07). These represent medium effect sizes [40]. Thus, occupation and tobacco consumption have a moderate influence on subjective auditory function.

The observation that socioeconomic and lifestyle factors predict one dimension of hearing function in middle age partially supports previous research [1,41], although notably Scholes et al. only found there to be a significant effect of socioeconomic status on hearing sensitivity for men and not women [41]. It is also important to consider that previous research has not applied Bonferroni correction to control for family-wise error rate, which may have resulted in an overestimation of reported effects [41]. Research has focused primarily on hearing sensitivity (as measured by pure tone audiometry) and not suprathreshold hearing including the perception of speech in noise, which may be more reflective of the daily demands of listening [1,41]. It may be the case that while socioeconomic and lifestyle factors impact hearing sensitivity and subjective hearing function, they do not impact speech perception.

However, the DiN task has been found to be less sensitive to hearing difficulty for those with mild hearing loss, where speech recognition remains intact [42]. The lack of an effect in the remote DiN task could be due to our sample being middle-aged, as a decline in speech in noise becomes most exaggerated from age 75 [43]. Furthermore, within our sample, the mean SNR-50 was −9.3 (SD = 4.37) which is indicative of normal hearing when hearing is measured using the DiN task [44]. In our sample, SNR-50 values ranged from −20.4 to 4, where a higher number represents worse performance. Armstrong et al. suggested that an appropriate cut-off level suggestive of insufficient performance would be > −5.55 to ≤ −3.80 dB and the optimal cut-off point for poor performance would be > −3.80 dB [44]. In our sample, 24 participants had insufficient performance, 30 participants had poor performance, and 154 had sufficient performance. A further complication of the DiN task is that using a closed set response such as numeric digits within a speech in noise task is that English digits 0–9 are distinct and would be easier to identify than more complex signals such as words/sentences; however, using numeric digits is more reliable for an online study as participants are able to report the digits they heard using a simple mouse click interface. The DiN task also did not include any familiarisation trials, so possible learning effects were not stabilised [27]. These factors may explain the absence of significant predictors of speech perception performance; primarily individuals with more severe hearing impairment, who might exhibit stronger associations with socioeconomic and lifestyle factors, were underrepresented.

The SSQ-12 mean was 7.47 (*SD* = 1.45), and scores ranged from 3.13 to 10, where a lower number reflects worse performance. Cañete et al. suggest, that when comparing the Spanish SSQ-12 to pure tone audiometry, an SSQ-12 score of ≤8.5 points indicates insufficient hearing [45]. Within our sample the mean SSQ-12 score is 7.47 which is considered reflective of hearing impairment. However, Cañete et al. did find significant differences between the mean SSQ-12 of the Spanish version and the English version, with non-hearing-impaired English speaking young adults (age range 18−29) from New Zealand having a mean SSQ-12 of 7.8 [45]. Due to scoring discrepancies between the Spanish and the English versions of the SSQ-12, it would not be prudent to categorise the hearing function of our sample based on SSQ-12 scores.

Tobacco consumption has been previously associated with hearing impairment when measured using pure tone audiometry to determine hearing sensitivity [12–16]. Similarly, occupation has been found to be associated with hearing sensitivity, with routine and manual occupations reported to have poorer hearing as measured by pure tone testing [1]. Digits in noise paradigms may be reflective of hearing sensitivity, as they have been found to significantly correlate with PTA [46–47]. The discrepancy between the lack of effect of tobacco consumption and occupation on hearing sensitivity (as measured by the DiN task) but the significant effect of these factors on subjective hearing ability (as measured by the SSQ-12) observed in this study was, therefore, unexpected. However, in mid-life, changes in hearing sensitivity may be more difficult to detect using the online DiN task employed in the present study. Measures sensitive to extended high-frequency hearing [48] or employing the antiphasic DiN [47] may be more sensitive. In contrast, we observed that increased tobacco consumption and routine and manual occupations were related to poorer self-reported hearing ability. It may be the case that the self-report measure taps into difficulty listening in real-world scenarios, which involves spatial hearing, informational masking, and listening effort. Such challenges may not be adequately captured by measures of hearing sensitivity, such as the DiN task or PTA [49–51]. As such, the discrepancy between the results of the speech perception models and subjective hearing ability models is likely due to the DiN task lacking sensitivity for detecting more mild hearing losses.

Recruitment via Prolific may have limited how representative the sample is, although a strength of this research is the recruitment of participants based on an equal distribution of income groups, which without the availability of Prolific’s filtered recruitment features, would have been difficult. However, Prolific requires consistent technological access, including access to a computer, Wi-Fi and online banking; these resources may not be available to everyone, particularly those in lower socioeconomic positions or people experiencing domestic or personal instability, such as living in temporary accommodation or having a disability. However, Prolific also has further beneficial characteristics for diverse recruitment such as flexible scheduling and home-based collection, meaning people who might otherwise be unable to get time off work to come to a laboratory or have caring responsibilities are able to take part in research. Furthermore, participants over 60 recruited from Prolific are found to have speech perception in noise abilities that do not significantly differ from participants of the same age tested in the laboratory [52].

The online nature of the study introduces some measurement concerns, particularly around the listening environment. While participants were instructed to use headphones, it was not possible to control for the sound output of different devices or any potential Wi-Fi connectivity issues that might have affected the quality of the DiN task. This could have introduced variability into the data, potentially explaining the lack of effect on speech perception. Although previous research has found that online versions of the DiN tasks correlate with DiN tasks conducted in a lab environment [53] and that SSQ-12 [26] scores correlated with remote DiN scores, this was not the case in our sample. We found no significant correlation between SNR-50 and mean SSQ-12, *r*(206) = −0.10, *p* = .173. It is important to note that Zadeh et al. conducted remote DiN tasks on a modest sample of participants (N = 34) who had been instructed by the researchers in person, so the findings may not apply to larger-scale online research, where there is an inability to communicate with participants directly [53]. For example, online participants might be less likely to adequately follow written instructions such as the requirement to be in a quiet environment, which may result in an elevated SNR-50 that is not consistent with their self-reported auditory function.

The study helps identify the period in the ageing trajectory at which socioeconomic and lifestyle factors may begin to impact hearing function. Mid-life hearing function within our sample appeared to be robust, with the majority of participants having an SNR-50 reflective of normal hearing. This was true among the diverse range of socioeconomic groups recruited (see Figs 2 and 5) which is a positive reflection on the maintenance of hearing in lower socioeconomic groups through mid-life, before a possible decline in later life.

Moving forward, it will be crucial to explore novel factors influencing speech perception ability, including diet, geographical location, and/or physical inactivity, which might better account for variability in hearing function. This study also lacks direct measurement of cardiovascular disease and health related factors, which may be direct physiological mechanisms underlying hearing function. The presence of cardiovascular risk factors, such as smoking, in those with hearing loss is well established [12–16], and our findings support this as tobacco consumption was a significant predictor of subjective auditory function in mid-life: smoking is a significant risk factor for cardiovascular disease [54] and cardiovascular disease is consistently associated with lower socioeconomic status [55]. It is important to identify factors predictive of poorer hearing function to establish preventive interventions and to encourage the uptake of hearing aids in at-risk populations, ultimately reducing hearing health inequalities as audiologists can target groups in most need. However, differences in individual characteristics, such as socioeconomic status, gender and ethnicity may not directly result in hearing loss but modify behaviour in a way that increases the risk of hearing loss. It may be the case that rather than targeting at-risk groups as to encourage audiological intervention, at risk groups in mid-life, for example smokers, should instead be provided with options to modify risks, such as harm reduction strategies like nicotine replacement therapy, or be given help to change the circumstances that led to dependency such as mental health distress. This layered approach acknowledging that people’s choices are constrained, might make way for meaningful interventions that makes taking care of hearing an option for everyone.

## Conclusion

This study aimed to explore the potential associations between socioeconomic, lifestyle, and demographic factors with midlife hearing function, focusing on both subjective auditory function (measured with the SSQ-12) and speech perception in noise (assessed using the DiN task). While the results did not reveal significant predictors for speech perception ability in midlife, the results did show significant predictors of subjective auditory function. Having a routine and manual occupation as compared to a managerial and professional occupation predicted worse self-reported hearing function, as did being a regular tobacco consumer when compared to non-consumers. Medium effect sizes were also observed for the occupation and tobacco consumption variables. These findings suggest that while there is some influence of lifestyle factors and socioeconomic factors on hearing function, their overall impact appears limited, within the midlife cohort examined, to subjective auditory function.

The lack of strong associations between these factors and speech perception ability could be attributed to several factors, including the relatively healthy hearing status of our sample and potential limitations in the online study design, which may have affected the reliability of the speech perception task.

This study offers valuable insights into midlife hearing function and provides a foundation for further research into novel predictors of hearing ability. Future studies should investigate additional factors that could influence speech perception ability and subjective auditory function, as well as recruit larger, more diverse populations to strengthen the generalisability of findings. Ultimately, identifying risk factors for poorer hearing function early in the ageing process is essential for developing preventive interventions and reducing hearing health inequalities across different socioeconomic groups. As hearing loss has a significant impact on quality of life, enhancing the understanding of predictors of hearing ability can guide audiologists and healthcare providers in targeting at-risk populations, thereby improving early intervention and access to hearing aids.

## References

[1] Tsimpida D, Kontopantelis E, Ashcroft D, Panagioti M. Socioeconomic and lifestyle factors associated with hearing loss in older adults: a cross-sectional study of the English Longitudinal Study of Ageing (ELSA). BMJ Open. 2019;9(9):e031030. doi: 10.1136/bmjopen-2019-031030 31530617 PMC6756470

[2] Tsimpida D, Kontopantelis E, Ashcroft DM, Panagioti M. Regional patterns and trends of hearing loss in England: evidence from the English longitudinal study of ageing (ELSA) and implications for health policy. BMC Geriatr. 2020;20(1):536. doi: 10.1186/s12877-020-01945-6 33319704 PMC7737370

[3] Rutherford BR, Brewster K, Golub JS, Kim AH, Roose SP. Sensation and Psychiatry: Linking Age-Related Hearing Loss to Late-Life Depression and Cognitive Decline. Am J Psychiatry. 2018;175(3):215–24. doi: 10.1176/appi.ajp.2017.17040423 29202654 PMC5849471

[4] Dawes P, Emsley R, Cruickshanks KJ, Moore DR, Fortnum H, Edmondson-Jones M, et al. Hearing loss and cognition: the role of hearing AIDS, social isolation and depression. PLoS One. 2015;10(3):e0119616. doi: 10.1371/journal.pone.0119616 25760329 PMC4356542

[5] Tsimpida D, Kontopantelis E, Ashcroft DM, Panagioti M. The dynamic relationship between hearing loss, quality of life, socioeconomic position and depression and the impact of hearing aids: answers from the English Longitudinal Study of Ageing (ELSA). Soc Psychiatry Psychiatr Epidemiol. 2022;57(2):353–62. doi: 10.1007/s00127-021-02155-0 34383085 PMC8784360

[6] Tsimpida D, Kontopantelis E, Ashcroft DM, Panagioti M. Conceptual Model of Hearing Health Inequalities (HHI Model): A Critical Interpretive Synthesis. Trends Hear. 2021;25:23312165211002963. doi: 10.1177/23312165211002963 34049470 PMC8165532

[7] Pierre PV, Fridberger A, Wikman A, Alexanderson K. Self-reported hearing difficulties, main income sources, and socio-economic status; a cross-sectional population-based study in Sweden. BMC Public Health. 2012;12:874. doi: 10.1186/1471-2458-12-874 23067045 PMC3533986

[8] Dawes P, Cruickshanks KJ, Moore DR, Edmondson-Jones M, McCormack A, Fortnum H, et al. Cigarette smoking, passive smoking, alcohol consumption, and hearing loss. J Assoc Res Otolaryngol. 2014;15(4):663–74. doi: 10.1007/s10162-014-0461-0 24899378 PMC4141428

[9] Rosenhall U, Idrizbegovic E, Hederstierna C, Rothenberg E. Dietary habits and hearing. Int J Audiol. 2015;54 Suppl 1:S53-6. doi: 10.3109/14992027.2014.972524 25549171

[10] Houston DK, Johnson MA, Nozza RJ, Gunter EW, Shea KJ, Cutler GM, et al. Age-related hearing loss, vitamin B-12, and folate in elderly women. Am J Clin Nutr. 1999;69(3):564–71. 10075346 10.1093/ajcn/69.3.564

[11] Halsted CH, Villanueva JA, Devlin AM, Chandler CJ. Metabolic interactions of alcohol and folate. J Nutr. 2002;132(8 Suppl):2367S-2372S. doi: 10.1093/jn/132.8.2367S 12163694

[12] Tang D, Tran Y, Dawes P, Gopinath B. A Narrative Review of Lifestyle Risk Factors and the Role of Oxidative Stress in Age-Related Hearing Loss. Antioxidants. 2023;12(4):878. doi: 10.3390/antiox12040878 37107253 PMC10135296

[13] Ciorba A, Chicca M, Bianchini C, Aimoni C, Pastore A. Sensorineural hearing loss and endothelial dysfunction due to oxidative stress: Is there a connection? J Int Adv Otol. 2012;8(1):16–20.

[14] Jamesdaniel S, Rosati R, Westrick J, Ruden DM. Chronic lead exposure induces cochlear oxidative stress and potentiates noise-induced hearing loss. Toxicol Lett. 2018;292:175–80. doi: 10.1016/j.toxlet.2018.05.004 29746905 PMC6131708

[15] Rivas-Chacón LDM, Martínez-Rodríguez S, Madrid-García R, Yanes-Díaz J, Riestra-Ayora JI, Sanz-Fernández R, et al. Role of Oxidative Stress in the Senescence Pattern of Auditory Cells in Age-Related Hearing Loss. Antioxidants (Basel). 2021;10(9):1497. doi: 10.3390/antiox10091497 34573129 PMC8464759

[16] Teraoka M, Hato N, Inufusa H, You F. Role of Oxidative Stress in Sensorineural Hearing Loss. Int J Mol Sci. 2024;25(8):4146. doi: 10.3390/ijms25084146 38673731 PMC11050000

[17] Phatak SA, Brungart DS, Zion DJ, Grant KW. Clinical Assessment of Functional Hearing Deficits: Speech-in-Noise Performance. Ear Hear. 2019;40(2):426–36. doi: 10.1097/AUD.0000000000000635 30134353

[18] Steptoe A, Breeze E, Banks J, Nazroo J. Cohort profile: the English longitudinal study of ageing. Int J Epidemiol. 2013;42(6):1640–8. doi: 10.1093/ije/dys168 23143611 PMC3900867

[19] Faul F, Erdfelder E, Lang A-G, Buchner A. G*Power 3: a flexible statistical power analysis program for the social, behavioral, and biomedical sciences. Behav Res Methods. 2007;39(2):175–91. doi: 10.3758/bf03193146 17695343

[20] Prolific [Internet]. 2014. Available from: www.prolific.com

[21] Office for National Statistics, International Regional and City statistics. 2021.

[22] Tsimpida D, Kontopantelis E, Ashcroft D, Panagioti M. Comparison of Self-reported Measures of Hearing With an Objective Audiometric Measure in Adults in the English Longitudinal Study of Ageing. JAMA Netw Open. 2020;3(8):e2015009. doi: 10.1001/jamanetworkopen.2020.15009 32852555 PMC7453309

[23] GSS Harmonisation Team. Ethnicity harmonised standard. 2011;1. https://analysisfunction.civilservice.gov.uk/policy-store/ethnicity-harmonised-standard/

[24] Office for National Statistics, Soc 2020 volume 1: structure and descriptions of unit groups. 2020.

[25] Office for National Statistics, Average household income, UK: financial year ending 2019. 2020

[26] Noble W, Jensen NS, Naylor G, Bhullar N, Akeroyd MA. A short form of the Speech, Spatial and Qualities of Hearing scale suitable for clinical use: the SSQ12. Int J Audiol. 2013;52(6):409–12. doi: 10.3109/14992027.2013.781278 23651462 PMC3864780

[27] Smits C, Theo Goverts S, Festen JM. The digits-in-noise test: assessing auditory speech recognition abilities in noise. J Acoust Soc Am. 2013;133(3):1693–706. doi: 10.1121/1.4789933 23464039

[28] Prolific. Prolific’s Attention and Comprehension Check Policy, [internet]. 2022 [updated 2022 November 21]. Available from: https://researcher-help.prolific.co/hc/en-gb/articles/360009223553-Prolific-s-Attention-and-Comprehension-Check-Policy

[29] Venables WN, Ripley BD. Modern Applied Statistics with S. 4 ed. New York: Springer; 2022.

[30] Booth DE, Gopalakrishna-Remani V, Cooper ML, Green FR, Rayman MP. Boosting and lassoing new prostate cancer SNP risk factors and their connection to selenium. Sci Rep. 2021;11(1):17877. doi: 10.1038/s41598-021-97412-2 34504230 PMC8429712

[31] Austin PC, Tu JV. Automated variable selection methods for logistic regression produced unstable models for predicting acute myocardial infarction mortality. J Clin Epidemiol. 2004;57(11):1138–46. doi: 10.1016/j.jclinepi.2004.04.003 15567629

[32] Aguinis H, Gottfredson RK, Joo H. Best-Practice Recommendations for Defining, Identifying, and Handling Outliers. Organ Res Methods. 2013;16(2):270–301. doi: 10.1177/1094428112470848

[33] R Core Team. R: A Language and Environment for Statistical Computing. Vienna (AT): R Foundation for Statistical Computing; 2025.

[34] Wickham H, Averick M, Bryan J, Chang W, McGowan L, François R, et al. Welcome to the Tidyverse. JOSS. 2019;4(43):1686. doi: 10.21105/joss.01686

[35] Wickham H, François R, Henry L, Müller K, Vaughan D. dplyr: A Grammar of Data Manipulation [Internet]. 2025. Available from: https://CRAN.R-project.org/package=dplyr

[36] Fox J, Weisberg S. An R Companion to Applied Regression [Internet]. 3rd ed. Thousand Oaks (CA): Sage; 2019. Available from: https://CRAN.R-project.org/package=car

[37] Arnold JB. ggthemes: Extra Themes, Scales and Geoms for ‘ggplot2’ [Internet]. 2024. Available from: https://CRAN.R-project.org/package=ggthemes

[38] Kassambara A. ggpubr: ‘ggplot2’ Based Publication Ready Plots [Internet]. 2023. Available from: https://CRAN.R-project.org/package=ggpubr

[39] Zhang D. rsq: R-Squared and Related Measures [Internet]. 2023. Available from: https://CRAN.R-project.org/package=rsq

[40] Cohen J. Statistical power analysis for the behavioral sciences. 2 ed. Hillsdale (NJ): Lawrence Erlbaum Associates; 1988.

[41] Scholes S, Biddulph J, Davis A, Mindell JS. Socioeconomic differences in hearing among middle-aged and older adults: cross-sectional analyses using the Health Survey for England. BMJ Open. 2018;8(2):e019615. doi: 10.1136/bmjopen-2017-019615 29391384 PMC5829909

[42] Dawes P, Fortnum H, Moore DR, Emsley R, Norman P, Cruickshanks K, et al. Hearing in middle age: a population snapshot of 40-to 69-year olds in the United Kingdom. Ear Hear. 2014;35(3):e44-51. 10.1097/AUD.0000000000000010 PMC4264521 24518430

[43] Dubno JR. Speech recognition across the lifespan: Longitudinal changes from middle age to older adults. Am J Audiol. 2015;24(2):84–7. doi: 10.1044/2015_AJA-14-0052 25767998 PMC4610266

[44] Armstrong NM, Oosterloo BC, Croll PH, Ikram MA, Goedegebure A. Discrimination of degrees of auditory performance from the digits-in-noise test based on hearing status. Int J Audiol. 2020;59(12):897–904. doi: 10.1080/14992027.2020.1787531 32673129

[45] Cañete OM, Marfull D, Torrente MC, Purdy SC. The Spanish 12-item version of the Speech, Spatial and Qualities of Hearing scale (Sp-SSQ12): adaptation, reliability, and discriminant validity for people with and without hearing loss. Disabil Rehabil. 2022;44(8):1419–26. doi: 10.1080/09638288.2020.1795279 32721200

[46] Schimmel C, Cormier K, Manchaiah V, Swanepoel DW, Sharma A. Digits-in-Noise Test as an Assessment Tool for Hearing Loss and Hearing Aids. Audiol Res. 2024;14(2):342–58. doi: 10.3390/audiolres14020030 38666901 PMC11047740

[47] Shehabi AM, Plack CJ, Zuriekat M, Aboudi O, Roberts SA, Laycock J, et al. Arabic Digits-in-Noise Tests: Relations to Hearing Loss and Comparison of Diotic and Antiphasic Versions. Trends Hear. 2025;29:23312165251320439. doi: 10.1177/23312165251320439 40116787 PMC11930467

[48] Helfer KS, Jesse A. Hearing and speech processing in midlife. Hear Res. 2021;402:108097. doi: 10.1016/j.heares.2020.108097 33706999 PMC7955108

[49] Fitzgerald MB, Ward KM, Gianakas SP, Smith ML, Blevins NH, Swanson AP. Speech-in-Noise Assessment in the Routine Audiologic Test Battery: Relationship to Perceived Auditory Disability. Ear Hear. 2024;45(4):816–26. doi: 10.1097/AUD.0000000000001472 38414136 PMC11175785

[50] Mecklenburg DJ, Graham PL, James CJ. Relationships Between Speech, Spatial and Qualities of Hearing Short Form SSQ12 Item Scores and their Use in Guiding Rehabilitation for Cochlear Implant Recipients. Trends Hear. 2024;28:23312165231224643. doi: 10.1177/23312165231224643 38361477 PMC10874150

[51] Sanchez-Lopez R, Dau T, Whitmer WM. Audiometric profiles and patterns of benefit: a data-driven analysis of subjective hearing difficulties and handicaps. Int J Audiol. 2022;61(4):301–10. doi: 10.1080/14992027.2021.1905890 33825590

[52] Shen J, Wu J. Speech recognition in noise performance measured remotely versus in-laboratory from older and younger listeners. J Speech Lang Hear Res. 2022;65(6):2391–7. 35442717 10.1044/2022_JSLHR-21-00557 PMC9567433

[53] Motlagh Zadeh L, Brennan V, Swanepoel DW, Lin L, Moore DR. Remote self-report and speech-in-noise measures predict clinical audiometric thresholds. Int J Audiol. 2025;64(6):618–26. doi: 10.1080/14992027.2024.2387291 39109478 PMC12951638

[54] Chen Z, Boreham J. Smoking and Cardiovascular Disease. Semin Vasc Med. 2002;02(3):243–52. doi: 10.1055/s-2002-35392 16222617

[55] Wang T, Li Y, Zheng X. Association of socioeconomic status with cardiovascular disease and cardiovascular risk factors: a systematic review and meta-analysis. J Pub Health. 2024;32(3):385–99. 10.1007/s10389-023-01825-4 PMC9867543 36714072

